# Supplementary material for: Predicting neural responses to intra- and extra-cranial electric brain stimulation by means of the reciprocity theorem
Source: PLoS Comput Biol. 2025 Dec 15;21(12):e1013782. doi: 10.1371/journal.pcbi.1013782 (PMC12704905; doi:10.1371/journal.pcbi.1013782)
Supplement: Attachment — Submitted filename: Response letter Reciprocity PLOS CB.pdf [file pcbi.1013782.s001.pdf]

Dear Editor;

We hereby resubmit our manuscript “Predicting neural responses to intra- and extra-cranial electric brain stimulation by means of the reciprocity theorem” by Torbjørn V. Ness, Christof Koch, and Gaute T. Einevoll for publication in PLoS Computational Biology.

We would like to thank the editor and the three referees for their thoughtful comments and efforts in reviewing the manuscript. In response to their criticisms, we have substantially revised and improved the manuscript. Among the major changes are

- a revised Fig 1 to illustrate how our work relates to previous applications of the reciprocity theorem (RT)
- a new section (Section 2.4) and corresponding new figure (Fig 5), demonstrating how the RT can be used with cells that exhibit intrinsic spiking behavior
- a substantially expanded discussion relating to the novelty and potential future application of the presented results.

Our responses to the reviewers are marked with blue text.

Changes in the manuscript are marked with red text.

With these changes we hope that the revised manuscript is found suitable for publication in PLoS Computational Biology.

Sincerely,

Torbjørn V. Ness

on behalf of the authors

Reviewer #1:

1. This manuscript presents an interesting and potentially valuable application of the reciprocity theorem to the field of electrical brain stimulation. The core idea – leveraging the RT to simplify and better understand the relationship between stimulation parameters and neuronal responses – is clear. The manuscript is generally well-written, and the authors demonstrate a good grasp of the relevant biophysical principles. I believe the work has the potential to be publishable in PLOS Computational Biology, but requires Major Revision to address several key concerns regarding rigor, clarity, and impact. The current draft leans towards being a methodological description with promising implications, rather than a conclusive study with significant new biological insights. Please find the point-by-point feedback below.

We appreciate that the reviewer recognizes the value of our manuscript. We agree that the manuscript primarily describes a new method with promising applications, rather than presenting new biological insights. We are currently applying the reciprocity-based approach to several ongoing projects with the aim of providing new biological insights into electric brain stimulation; the aim of the current manuscript is, instead, to thoroughly present the reciprocity-based method itself. This will allow subsequent applications of this approach, by us and others, to more cleanly focus on biology.

We would, however, like to point out that explaining the results of Lee et al. (2024), and providing power-laws for the distance-dependence and frequency-dependence of the response of neurons to electric stimulations do constitute important biological insights.

2. Relation to the Biological Problem: The authors utilized a largely passive (non-spiking) single-cell model, representing the simplest possible case and one well-explored in the literature. The next steps for such modeling remain unclear. Traditionally, this would involve embedding a single cell into a spiking network representing a cortical column or brain region. However, the feasibility of this approach is uncertain, as network dynamics can quickly become chaotic and non-linear. The authors should consider whether their approach has a future in addressing more realistic, network-level modeling.

As the reviewer points out, the current manuscript focuses on the subthreshold single-cell response of electric stimulation, and the natural next step will be to extend the simulation to a spiking network. In response to the referee's comment, we added a new Section 2.4 and a new Figure 5 to better explain how the RT-based approach can be used to address the effect of ES at the network level, line 284:

"2.4. *Intrinsically active cells*

We demonstrated that the RT-based approach works well in the approximately linear subthreshold regime (Fig 2), however, neurons are—due to their ability to fire action potentials—fundamentally non-linear entities. Some classes of neurons also exhibit intrinsic spiking activity, like in the subthalamic nucleus (STN) of the basal ganglia which is an important target for DBS therapy in patients with Parkinson's disease [75].

Note that even though the effect of ES on neural dynamics can be non-linear, the direct, isolated effect of ES on membrane potentials usually is linear: As in the traditional way of simulating, the ES enters as an additive term in the cable equation, $(1/r_i)\partial E_{||}(x, t)/\partial x$,

which only depends on the electric field along the neurite $E_{||}$ and the cable properties, and not on the membrane potential. The ES-induced effect itself on the membrane currents is therefore linear and additive, even when the resulting membrane potential and network dynamics is not. The RT-based approach might therefore be able to isolate the linear effect of the ES itself on the membrane potential, which can then be added to a non-linear model.

To investigate this, we considered a macaque STN neuron (Fig 5A), which intrinsically spikes at around 30 Hz (Fig 5B). We introduced an ES with parameters arbitrarily chosen to have an amplitude of 100 μA at 10 Hz, located 500 μm away from the soma (Fig 5C). The spiking behavior of the cell was substantially modulated by the ES (Fig 5D versus Fig 5B). Since the behaviour of the membrane potential is clearly non-linear, the RT can not be used directly; however, the effect of an ES can still be estimated, by inserting the ES current (Fig 5C) into the soma of a passive version of the cell model, and calculating the resulting V_e at the stimuli location: Through the RT, this corresponds to the isolated ES-induced response of the somatic membrane potential ΔV_m (Fig 5E). We then introduce this effect into the active intrinsically spiking cell model (instead of the ES) as the somatic current input that in isolation would cause this ΔV_m. This gave rise to a very similar ES-modulated activity as the traditional approach (Fig 5F versus Fig 5D).

The example above illustrates that the effect of the ES can——at least in this case——be isolated and then added to a non-linear model. This is relevant for point neuron simulations of deep brain stimulation of the basal ganglia: Through the RT and using biophysically detailed cell models, we can efficiently and easily estimate how different cell-types at different locations are affected by ES. The non-linear effect of the ES at the network-level (with intrinsically spiking neurons) can then be investigated by highly efficient point-neuron, or potentially even mean-field network models."

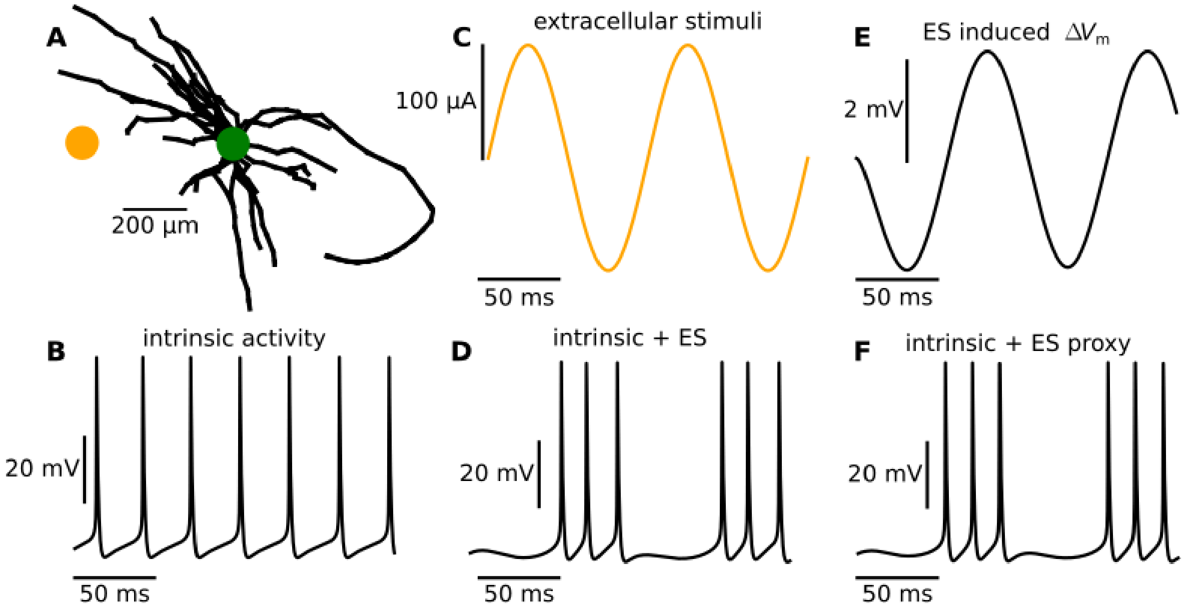

Fig 5: Intrinsically active cell model. A: Macaque STN neuron from Miocinovic et al. (2006) [43]. **B:** The intrinsic spiking activity of the model in the absence of input. **C:** An electric stimulation (location marked by orange dot in panel A) at 10 Hz with a current amplitude of 100 μA, 500 μm away from the soma of the cell model. **D:** The somatic membrane potential of the cell under the influence of the ES in panel C, simulated through

the traditional approach. **E:** Through the RT, we can calculate the effect of the ES in isolation, by inserting the current in panel C into the soma of a passive version of the cell model, and calculating V_e at the ES location. The resulting amplitude of V_e was 2.1 mV. From the same simulation, we can calculate the input impedance of the model, and from this the equivalent somatic current amplitude of 67.6 pA needed to cause this 2.1 mV oscillation. **F:** The original active cell model is stimulated with an "ES proxy", that is, by injecting this current into the soma (amplitude 67.6 pA) designed to mimic the ΔV_m in panel E. This closely approximates the direct simulation (panel D).

We also expanded the discussion of this in the "Outlook" section, line 562:

"To study the effect of ES at the network level, one can, in principle, use large-scale network models of biophysically detailed multicompartment cell models, and simulate the ES with traditional methods. However, due to their associated high computational demands, such models are challenging to work with. Furthermore, comprehensive parameter scans are not within reach.

A common way to study network effects is via point-neuron networks or mean-field models [118], including the effects of ES/tES [103; 106; 119–122]. However, the effects of ES are mediated by electric potential gradients over neural morphologies, which are not part of such approaches. It is here that the RT-based approach can be used to estimate the somatic membrane potential response to ES with biophysically detailed cell models, which can then be added to the membrane potential of simplified neuron models [123] or point neurons in a network model.

The assumption of linearity is therefore not as restrictive as it might first seem: The RT-based approach is highly accurate in estimating the effect of ES in the subthreshold regime (Fig 2E, Fig 5F, Fig 6K), and the point-neuron models account for the non-linear spiking behavior. The RT-based approach incorporates the detailed cellular morphology, and can therefore be expected to be more accurate than approaches using simplified or reduced morphologies for capturing the effect of ES. Furthermore, it will be more computationally efficient, since the network activity can be simulated with point-neurons rather than neuron models having a spatial structure. The RT-based approach can directly predict the somatic V_m effect of ES throughout a volume, for different cell types, and under different stimulation parameters, which can then be combined with point-neuron network simulations using NEST [124], or even whole-brain mean-field models using The Virtual Brain [125]."

We would also like to add that we are currently using the reciprocity-based method with spiking point-neuron network models ourselves, and are furthermore engaged in discussions with experts in mean-field modelling regarding how we can apply the reciprocity-based framework in the mean-field model context. This is, however, beyond the scope of the present manuscript.

3. Limited Biological Validation & Scope: The manuscript heavily relies on simulations. While simulations are essential, the lack of direct comparison to a wider range of experimental data, beyond the reference to Lee et al. (2024), significantly weakens the impact. The current analysis appears largely hypothetical. Where are the predictions that differentiate this approach from existing modeling strategies and can be experimentally tested? A strong

addition would be a validation against a broader set of established experimental paradigms, or a demonstration of a novel prediction that can be directly tested.

As the reviewer mentions, we compare our simulation results to experimental microstimulation data from Lee et al. (2024), and find an excellent match (line 141). Furthermore, we report excellent agreement with experimental data regarding tES (line 395). Apart from these examples, however, not many experimental studies have reported intracellular responses to extracellular stimulation, as such measurements are very challenging to do.

As we point out, both the traditional as well as the reciprocity-based methods give equivalent numerical predictions in the linear regime. That is, if properly implemented, there should not be any discrepancy between the two. The value of the reciprocity-based approach is that it provides an entirely new perspective which can be helpful for generating novel insights: For example, we predict that the frequency- and cell-type independent subthreshold effect of electric stimulation reported by Lee et al. (2024) will only hold for near-by ES below about 100 Hz (around line 442).

See also our response to the next point.

4. Validation vs. Verification: On a related note, the authors claim to have “validated” the approach by comparing simulations against other simulations, which is an overreach. True validation requires generating testable predictions and experimentally validating them. What the authors have performed is more accurately described as a sanity check or a first approach verification.

We thank the reviewer for bringing up this important point. In the manuscript, we only aim to show that our approach gives numerically equivalent results to the traditional approach, validating the reciprocity-based approach by comparing it to the traditional approach. The latter is grounded in the community’s biophysical understanding of neurons and electrical properties of brain tissue, and is underpinning a vast amount of research (see Aberra at el. *bioRxiv* (2025) for a recent example of model validation against experimental data).

We added a brief discussion regarding this to the “Limitations” section, line 550:

“We found good agreement between our simulations and experimental data from microstimulation (Sec 2.2.1) and tES (Sec 2.6). The main validation of the RT-based approach is, however, the demonstration that it provides results equivalent to the traditional approach to simulating brain stimulation in the subthreshold regime. The validity of the reciprocity-based approach is therefore anchored to the traditional approach, which has been rigorously validated (see Aberra et al. (2025) for a recent example).”

5. Overstatement of Novelty: The authors present the application of the RT to this specific context as novel. While the application of electrical stimulation is relatively unexplored, the RT itself is well-established and used in both EEG and brain stimulation (e.g., for stimulation electrode location optimization). Furthermore, numerous preceding works exist that

generated similar insights into single-cell behavior due to electric current/field injection. The authors present no clear reason to prefer their implementation over preceding approaches. The introduction needs to better acknowledge the existing use of the RT in related neurophysiological modeling. The authors should be more specific about what is novel here – a particular application of the RT to single-cell modeling.

This is yet another important point, and a clearer explanation is warranted. As the referee points out, the RT has been previously used in the context of electric brain stimulation (e.g., "stimulation electrode location optimization"). Further examples in the context of optimization of electric brain stimulation are (the red entries were not previously cited in our manuscript):

- Huang, Y., Parra, L. C., & Haufe, S. (2016). The New York Head—A precise standardized volume conductor model for EEG source localization and tES targeting. *NeuroImage*, *140*, 150–162. https://doi.org/10.1016/j.neuroimage.2015.12.019
- Dmochowski, J. P., Koessler, L., Norcia, A. M., Bikson, M., & Parra, L. C. (2017). Optimal use of EEG recordings to target active brain areas with transcranial electrical stimulation. *NeuroImage*, *157*(May), 69–80. https://doi.org/10.1016/j.neuroimage.2017.05.059
- Wagner, S., Lucka, F., Vorwerk, J., Herrmann, C. S., Nolte, G., Burger, M., & Wolters, C. H. (2016). Using reciprocity for relating the simulation of transcranial current stimulation to the EEG forward problem. *NeuroImage*, *140*, 163–173. https://doi.org/10.1016/J.NEUROIMAGE.2016.04.005
- Fernández-Corazza, M., Turovets, S., & Muravchik, C. H. (2020). Unification of optimal targeting methods in transcranial electrical stimulation. *NeuroImage*, *209*, 116403. https://doi.org/10.1016/J.NEUROIMAGE.2019.116403
- Gross, J., Junghöfer, M., & Wolters, C. (2023). Bioelectromagnetism in Human Brain Research: New Applications, New Questions. *Neuroscientist*, *29*(1), 62–77.

However, these and other papers use the RT to link *extracellular* current sources to *extracellular* potentials (or sometimes current dipoles to electric fields). This is essentially pure physics, assuming a linear Ohmic extracellular medium. The neurons themselves are not considered. Thus, to optimize electrode location, the electric field in a region of interest is maximized. For example, if a dipole at a certain location and orientation causes a strong EEG signal at a given electrode, the standard application of the RT (see Rush and Driscoll et al. 1969) implies that stimulating through this electrode will cause a strong electric field at the location of the dipole. However, this approach does not consider how the electric field affects neurons.

What we point out is that one can directly account for the effect of the stimulation *on the somatic membrane potential* by moving the current source into the soma. For electrode location optimization, the RT principle allows us to directly maximize the membrane potential response of any one cell-type.

This is an important difference: While it is fair to assume that the neural effect will be correlated with the electric field strength, the field strength itself is not very informative about, for example, what cell-type of neurons are most affected, how strongly they are affected, and what is the optimal stimulation frequency to use. Linking the stimuli directly to membrane

potential responses is therefore an important advantage, since it is the effect on neurons we are ultimately interested in.

We clarified this in the revised manuscript (line 52):

“The RT has been used in the context of optimizing electric brain stimulation, in particular for tES [52-56]. Importantly, however, these previous usages of the RT did not explicitly consider neurons or their properties, only the neural tissue as a whole (Fig 1A,B).”

Around line 438, we added:

“..., and extracellularly measured potentials, without explicitly considering the neurons themselves. We here show that by placing an electrode in the soma of a neuron model, we can use the RT to directly study ES.”

Around line 475 we added:

”Note that the RT has been used for optimizing tES with the goal of maximizing the electric field at a given location [52-56]. However, these studies did not use intracellular currents, and were therefore, in practice, agnostic to the neurons and their properties themselves. This approach does not, for example, distinguish between tDCS and tACS since the spatial properties of the induced electric fields are typically assumed to be frequency-independent. However, tDCS and tACS can be expected to affect neurons differently. The possibility to directly optimize for a cell-type specific V_m response at a given frequency via the RT is a key step towards more controllable tES. ”

We also modified Figure 1 to illustrate this difference.

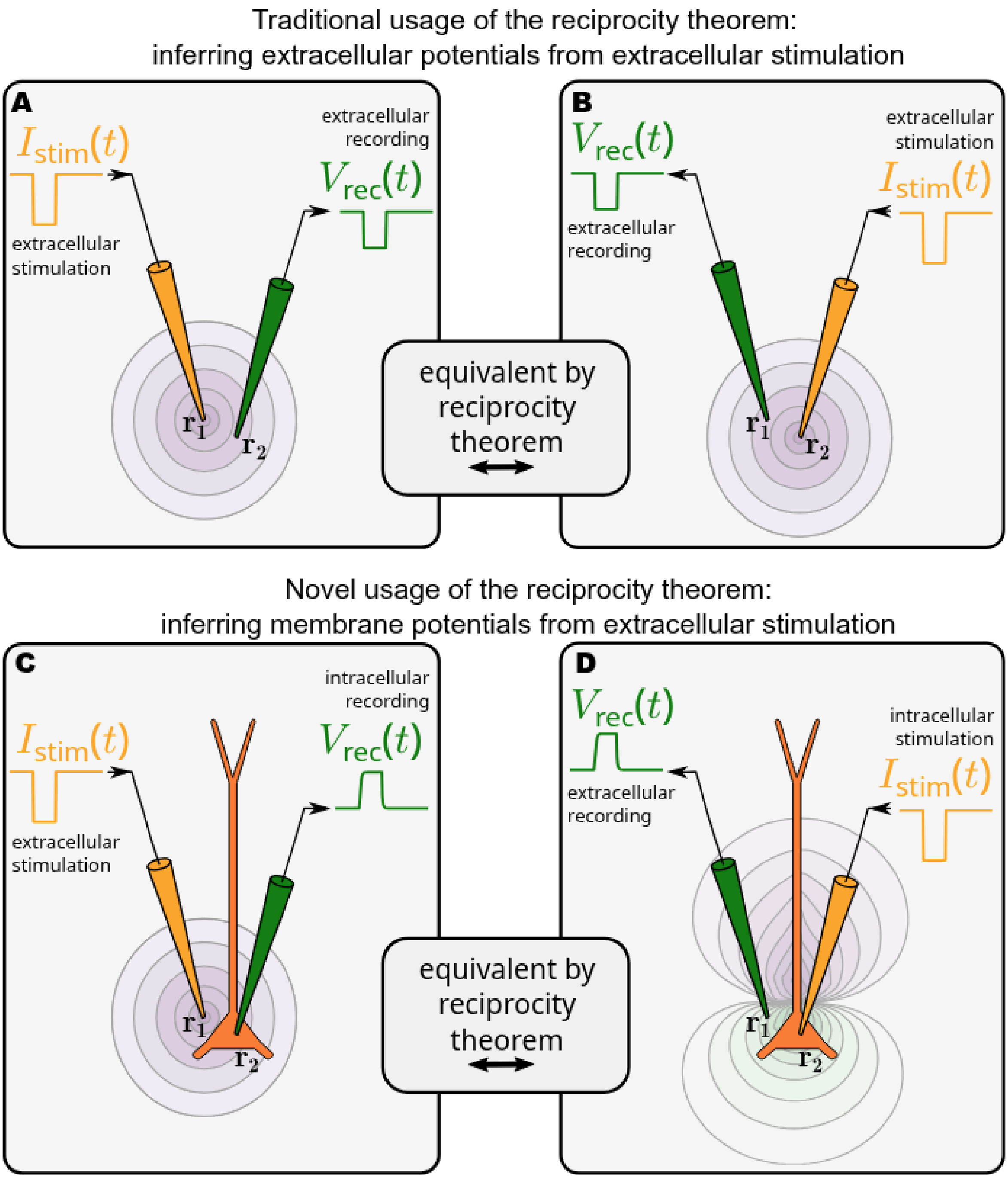

Fig 1: The reciprocity theorem in the context of electric brain stimulation. A, B: The traditional usage of the RT is to link extracellular current sources to extracellular potentials (or sometimes current dipoles to electric fields), without explicitly considering neurons and their properties [52–56]. **C, D:** We here point out that by moving the current source into the cell, the RT can be used to directly account for the effect of ES on membrane potentials.

6. Clarity of Practical Implications for tES: While the manuscript highlights the potential to predict somatic membrane potential changes due to tES, the connection to real-world scenarios is weak. For example, the authors injected the electric current at the soma of their

model – in reality, the electric field is much larger than the neuron and affects all compartments simultaneously.

As the reviewer points out, while stimulating, the electric field affects all compartments of a neuron simultaneously. This is one of the reasons why the effect of electric stimulation has been difficult to intuitively understand. However, the advantage of the reciprocity-based approach is that it does, in fact, take into account the entire morphology, even though the current is injected only into the soma. The resulting membrane currents are distributed across the morphology of the cell. From these distributed membrane currents we can calculate the EEG signal (based on methods, for example, presented by Næss et al. (2021)). The reciprocity theorem ensures that if the current was injected instead through the EEG electrode, the resulting somatic membrane potential response is the same as the originally calculated EEG signal. Note also that we tested this approach against the traditional method by applying the quasi-uniform approximation (see Figure 5K and Bikson et al. (2013); Aberra et al. (2023)).

7. Insufficient Justification for Specific Parameters/Models: The choice of specific cell models (rodent's L5 pyramidal and interneuron, an axon) feels somewhat arbitrary. What is the rationale behind this selection, and how generalizable do the authors expect the results to be to different neurons of different species (particularly, humans)? A more systematic exploration of the impact of different cell morphologies and biophysical properties on the results would strengthen the conclusions.

We agree that the chosen cell models are somewhat arbitrary. We chose the rodent L5 pyramidal cell from Hay et al. (2011) for its prominent role as a "default" pyramidal cell model for computational research in the field. We contrasted these results with results from an example interneuron model, to demonstrate how the reciprocity-based approach can highlight cell-specific features of electric stimulation. Furthermore, axons are expected to be the most sensitive neural element to electric stimulation (confirmed by our simulations), making them highly relevant for our study.

We chose representative exemplar cell models rather than scanning through data-bases of cell models in different brain regions in different species in order to keep the analysis clean and focused on presenting the reciprocity-based approach to brain stimulation. A comprehensive investigation of different cell types in different species is worthy of an independent study, and we are indeed currently pursuing this in a separate study. However, we know of no reason that the biophysical principles described here, and tested on rodent neurons, will be substantially different when applied to non-human primate or human neurons.

The reciprocity theorem itself is, of course, independent of the choice of cell model as long as the behavior is approximately linear. We now added a brief comment on this under "Limitations" line 544:

"In this study we were primarily concerned with demonstrating how the RT can be used to understand ES, and our results are therefore more illustrative than comprehensive. We only tested a few cell models from the literature. Further studies should scan through entire cell databases from ModelDB [115], The Allen Brain Institute, or BBP [27; 45; 116; 117], to

demonstrate the applicability of the same principle to neurons of different cell types and brains of different species, which will be important for better understanding ES. ”

Reviewer #2:

In “Predicting neural responses to intra- and extra-cranial electric brain stimulation by means of the reciprocity theorem” (PCOMPBIOL-D-25-01091), Ness et al. use the "Reciprocity Theorem" to predict the effects of electrical brain stimulation, and in particular, transcranial electrical stimulation.

The reciprocity theorem is a physical principle that allows one to exchange the location of current and voltage measurements within (most) circuits. Although this theorem is fairly straightforward—and used extensively in other fields, it has seen limited use in neuroscience and this is the first time I have seen it used to explore brain stimulation. Except for a few points, below, the paper generally seems technically sound. It is extremely clearly written with lovely illustrations.

We are happy to hear this.

1. Its major weakness is that I’m not sure what new insight this method adds for practitioners of brain stimulation. The major conclusion (§3.3, 3.5) is that the depolarizing effects of transcranial electrical stimulation are far too weak to directly drive spiking. This is already the overwhelming consensus from computational and experimental studies; indeed, the field has struggled too how that The real mystery, as the authors note on p. 15, is how network mechanisms translate these small polarizations into surprisingly robust changes in (e.g.,) spike timing seen in animal models and BOLD. The paper briefly touches on this at the top of p. 14-15. If the authors could expand on this and offer candidate mechanisms, it would be a really impactful paper. Otherwise, it’s essentially a editorial judgement: this uses a standard (but under-appreciated) to re-confirm widely accepted results, but it is an interesting perspective and does tie together aspects of the field that may not be obviously related.

We agree with the referee that our result, demonstrating that the effect of tES is too weak to directly drive spiking, is not novel, but is consistent with a large body of previous research. We modified the text in “3.3. Transcranial electric stimulation” to make this clearer (line 498):

“Our results are consistent with the consensus that observed effects of tES in humans must come about indirectly, either by enhancing spike synchrony in specific frequency ranges or from mechanisms like stochastic resonance [11; 30; 33; 34].”

We believe that the reciprocity-based approach can be helpful for studying candidate mechanisms for how tES affects neural activity. Accordingly, we substantially expanded the discussion of this in the manuscript, see also our response to point #2 from Reviewer #1. Indeed, we currently are pursuing such a project with a point-neuron network, via the methods suggested in “Outlook”, but this is outside the scope of the current manuscript.

Finally, we would like to emphasize that our manuscript goes beyond studying tES to explain the surprising cell-type and frequency-independent results for nearby ES presented by Lee et al. (2024), and predicts that these results are expected to hold only for nearby neurons below about 100 Hz. Furthermore, we supply analytic formulas and power laws for both the

frequency-response and distance-dependence of ES, which has, as far as we know, not been reported previously.

Minor:

2. The authors do an excellent job discussing several papers that (in my opinion), are both influential and often misinterpreted. The discussion of Grossman et al. (2017) should probably also reference Rampersad et al. (2019; Neuroimage), who re-modelled the mouse and found that the electric field was very, very strong (300+ V/m); note that Grossman also removed the skin, which removes a lot of the shunting. I think that, rather than the current, ought to be the focus. I don't have much to add re: the 1 V/m but thank you for including that nuance — those early experiments conflate the (true) presence of an effect with its detectability. I would note that other estimates of the E-field in human brains suggest that up to 0.8 V/m (at 2 mA) is achievable in humans (Huang et al, 2017; ELife—but note the correction).

We thank the reviewer for this helpful comment, and for these relevant references that we had missed. They have now been added at multiple locations to the revised manuscript.

3. Temporal interference needs to be discussed carefully since there is not yet a consensus on how it acts. In the Mirzakhalili model, the demodulation is only partially effective; this is consistent with data on amplitude-modulated tACS. Are the authors sure this isn't important? Can it somehow be addressed via RT or does it provide more of an upper bound?

This is an important and interesting question. In the Mirzakhalili model, the demodulation happens because the high-frequency stimulation causes more sodium than potassium activation. In essence, the prediction from this is that TI will lead to a conduction block in neurons outside of the target region (the persistent high-frequency stimulation causes a persistent activation of sodium, leading to a conduction block), while the firing rate of neurons inside the target region will follow the envelope frequency.

We are able to reproduce this effect using the traditional modeling approach. However, this requires extremely high-amplitude fields, far outside of what can be expected in humans (the TI-induced membrane potential oscillations needed to be on the order of 10 mV). For more clinically relevant low-amplitude fields (membrane potential oscillations on the order of 10 μV), this mechanism does not work, since neither the sodium nor potassium currents are sufficiently activated by the stimulation. Essentially, the cell models respond as if they are passive, and as pointed out by Mirzakhalili et al., the demodulation requires some sort of non-linearity to work.

An alternative mechanism which can provide the non-linearity needed for the demodulation is the spiking threshold itself, e.g., via stochastic resonance. We are currently investigating this via the reciprocity-based approach to predict the somatic membrane potential response from a given TI, using this estimate to drive point neurons in a network model. The preliminary results are encouraging, and demonstrate that the RT can be used to study TI, at least in this context. That project is beyond the scope of the present study, however.

We revised the discussion regarding this point, in section 3.4:

"Mirzakhalili et al. (2020) [31] demonstrated how an ion-channel-mediated rectification mechanism could explain how neurons respond to the envelope frequency during TI stimulation. It is plausible that such non-linear effects are needed to explain how spikes are directly evoked by extremely high-amplitude temporal interference stimulation, such as described by Grossman et al. (2017) [97] (see also discussion in Vieira et al. (2024) [33]). If so, the RT-based approach is not suited to study this phenomenon. How weak tES-mediated V_m perturbations affect neural networks, on the other hand, might be easiest to understand through simplified network simulations, where the RT-based approach can be useful (see Outlook)."

4. On p. 3, it notes that the RT is applicable in the subthreshold regime and this approach apparently requires an ad hoc scaling factor (§4.7) to keep the model there. It feels a little circular to me to make this adjustment and then conclude that the effects, assumed to be small, are in fact small. Is there a principled way to break this loop or a diagnostic that tells you when you're in trouble? In other words, suppose I want to simulate a 15 mA current pulse? Is it really supra threshold or have I mischosen the scale factor?

We understand that this point requires a better description. The relevant diagnostic is not the amplitude of the extracellular tES current but whether the associated membrane potential remains in the subthreshold regime.

If we want to know the membrane potential response to a 15 mA tES at a given scalp location, we use the RT as applied in Fig. 5 (whereby a 1 mA tES gives rise to a 0.038 uV membrane potential) and scale it up by x15. That is, a 15 mA tES changes the membrane potential by 0.57 mV (see the simulation in Fig. R1 below). This remains completely within the linear, subthreshold regime of the cell and we can expect the result of applying the RT principle therefore to be accurate. When injecting a 20-fold larger current of 300 mA (do not do this at home!), the linear RT approach predicts a membrane potential of 20*0.57 mV = 11.4 mV. In Figure 2 in the manuscript, the cell starts to behave non-linearly when the V_m-response is ~10 mV (which we can predict will occur for a tES amplitude of ~260 mA). That is, the inferred V_m-response is beyond the range of applicability of the RT principle for this cell. The diagnostic is that the membrane potential response to tES remains below a few millivolts.

Switching to active cell models (as in Figure 2), introduces the complication that the linear regime of the membrane potential response to intracellular and extracellular currents are vastly different. As pointed out above, the pyramidal cell model we used responds linearly for tES amplitudes below about 260 mA. For intracellular currents to the active Hay model, however, the amplitude must instead be below about 200 pA. An ES current amplitude which results in a subthreshold V_m-response will often give a suprathreshold response if given intracellularly, and the RT-based approach would not work.

To address this methodological difficulty, we use a fixed subthreshold current amplitude for the intracellular current to active cell models, and scale the resulting V_e to represent the extracellular current amplitude. Note that the "target signal"—that is, the membrane potential response of the active cell model to ES simulated through the traditional approach—was not scaled.

To summarize: There are no constraints per se on the allowable magnitude of stimulation current for the RT-approach to be applicable. The only relevant constraint to this approach is, as stated above, that the ES induced V_m-response should be below a few millivolts so that the resulting membrane-potential response is linear, as demonstrated in Figure 2 in the manuscript.

We extended the description of this, line 111:

"That is, while ES is applied directly to the fully active cell model, the reciprocity-based approach is a linear extrapolation of the subthreshold response of the active cell model (see Methods). This approach can be expected to be accurate when the membrane potential response from ES is within the linear regime. Indeed, the active cell model effectively responds linearly to ES, …"

We also added in the Methods section, line 663:

"The current amplitudes used in different types of ES vary over many orders of magnitude, from microampere or less in microstimulation, to milliampere in tES. The scaling factor varies by the same amount. Note, however, that this is unproblematic as long as the membrane potential response of the neuron to ES is in the linear, *i.e.*, subthreshold regime, in practice below a few millivolts (Fig 2). This is what matters, not the amplitude of the tES."

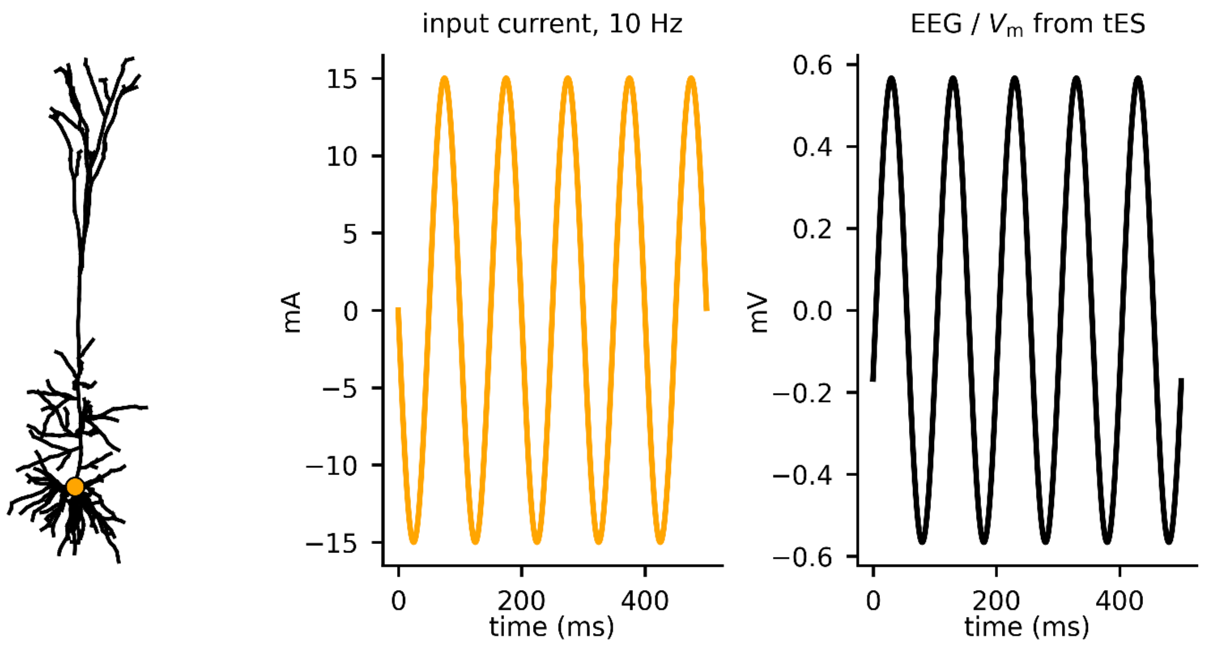

Figure R1: Effect of 15 mA tES. *The EEG response from a 15 mA somatic current input, and hence the membrane potential response from a 15 mA tES, has an amplitude of 0.57 mV, which is in the linear subthreshold regime. As long as the V_m response to ES is below a few millivolts,*

we can trust the RT to be accurate, regardless of the amplitude of the current itself. The simulation is otherwise identical to that of Figure 5 in the manuscript.

5. Both Aberra et al. and Vieira et al. have recent preprints examining the effects of electric fields on axons, which may be of interest to the authors.

We thank the reviewer for pointing us to these two relevant papers:

- Aberra, A. S., Miles, M. W., & Hoppa, M. B. (2025). Subthreshold electric fields bidirectionally modulate neurotransmitter release through axon polarization. *BioRxiv*, 2025.02.22.639625. https://doi.org/10.1101/2025.02.22.639625
- Vieira, P. G., Krause, M. R., Laamerad, P., & Pack, C. C. (2025). Brain stimulation preferentially influences long-range projections. *BioRxiv*, 2025.02.19.639189. https://doi.org/10.1101/2025.02.19.639189

The preprint from Aberra et al. (2025) is now cited at various locations in our manuscript, and in addition we mention it, line 225:

"Axon terminals are known to be the neural element with the strongest membrane potential response to ES [26; 27; 72]; indeed, it has recently been demonstrated that subthreshold polarization of axon terminals can strongly affect neurotransmitter release [46]. It is therefore often of interest to know the V_m response of axons in response to ES or tES. "

The preprint from Vieira et al. (2025) is mentioned, line 202:

"To give an example of how this insight might be useful: Vieira et al. (2025) [71] demonstrated that a small subgroup of cells were particularly strongly affected by tACS. Our results predict that this subgroup of neurons should also exhibit particularly strong current-dipole moments from somatic current input, which is straightforward to simulate. This can potentially be used to identify this particularly tACS-sensitive population by scanning through data bases of cell models. "

6. Section 2.4 argues that implants may boost electric fields. The authors might consider discussing Mercadal et al. (2022; J Neural Engineering), who argue that metal is less of a problem than believed because of the mismatch between ionic and electronic current flow? More generally, this section is interesting but needs to be tied more tightly to the paper's message. Is the argument that ECOG recordings overstate ||E||?

This relevant reference is now cited around line 339. The main motivation for Section 2.5 (previously Section 2.4) is not to give a review of the effect of implants on ES, but rather to demonstrate how one can, via application of the RT, obtain additional insights from previous studies. To make this motivation more clear we added (line 321):

“In this subsection, we demonstrate how we can use the RT for more complex extracellular mediums, and how to leverage the RT to gain additional insights into ES from previous research that explored extracellular potentials.”

With regard to ECoG recordings, it has previously been demonstrated that if the exposed cortical surface is covered by a non-conducting material (like a mineral oil), the extracellular potential (stemming from neural activity) will be larger than if the surface cover is more conductive, like saline [Pettersen et al., 2006; Rogers et al., 2020; Halnes et al., 2024]. It is not the case that ECoG recordings overstate the extracellular potential; rather, the extracellular potential at the cortical surface is genuinely increased by the presence of the non-conducting cover material. Through the RT, this implies that the somatic membrane potential response of neurons in response to an ES at the cortical surface will also be increased if the cortical surface is covered by a non-conducting material.

Reviewer #3:
In their work Ness et al. present applications of the reciprocity theorem for invasive and non-invasive electric brain stimulation to predict neural responses to stimulation. It leverages the principles of RT to infer how extracellular currents (induced by stimulation) affect intracellular potentials affecting neural behavior.
The manuscript is very well written, and provides interesting insights to the cellular effects of electric stimulation. I generally support publication of the manuscript, I only have a few remarks on the presentation of the work.

We thank the reviewer for the favorable description of our manuscript.

1. I am missing a description of the putative mechanisms of ES/tES (tDCS/tACS) in the introduction. All the authors mention so far is that these mechanisms of tES are controversial. However, especially at the cellular level there are many studies demonstrating effects electric stimulation (Bindman et al., 1964; Fröhlich & McCormick, 2010; Krause et al., 2019; Ozen et al., 2010; Reato et al., 2010).

This was not our intention. We now clarified that the biophysics of ES itself is well understood. We do not think it is within the scope of the current manuscript to go into detail about the mechanisms underlying ES, as there are many excellent sources for this (line 33):

"The basic biophysical mechanism underlying ES in the low kHz range is well described in the literature [36–41]: A spatial gradient in the electric potential outside a neuron's morphology will affect the neuron's membrane potential. Given the known biophysics it is, in principle, straightforward to estimate the effect of ES via computational modeling [27; 31; 42–46]."

2. With respect to the implications of the work for tES the manuscript has a strong focus on temporal interference stimulation (TI), which is a very novel technique. I think it is very fair to discuss it here, but I think the authors should also go into a bit more depth in terms of the implications of their work for more established tES techniques (i.e. tDCS and tACS).

We modified the revised manuscript to more generally address tES, and added a short paragraph regarding implications for future studies of tDCS and tACS (line 475):

"Note that the RT has been used for optimizing tES, where the goal has been to maximize the electric field at a given location in the brain [52-56]. However, these studies did not use intracellular currents, and were therefore in practice agnostic to the neurons and their properties themselves. This approach does not, for example, distinguish between tDCS and tACS since the spatial properties of the induced electric fields are typically assumed to be frequency-independent. However, tDCS and tACS can be expected to affect neurons differently. The possibility to directly optimize for a cell-type specific V_m response at a given frequency via the RT is a key step towards more controllable tES. "

3. There are some constraints in using computational headmodels like the NY head in estimating electric fields in the brain that should be mentioned. These models have to make assumptions about tissue conductivities and limit the number of compartments the head is segmented into which will affect the resulting electric field strength and distribution. This introduces uncertainty in the estimations of induced electric fields the authors discuss. I

would also recommend adding a brief description of the NY head (number of compartments, assumed tissue conductivities).

We now added a brief discussion of the uncertainty in the estimations (line 395):

“Note, however, that even though detailed head models have been demonstrated to be quite accurate for calculating electric fields from tES [93], there is considerable variability in both the anatomy of individual human brains, and in their conductivity values. The electric fields from tES will therefore also be person-specific [96]. The numbers presented here should therefore be viewed only as plausible examples.”

We also added a better description of the NY head model (line 621):

“Since the tES electrodes are far away relative to the size of neurons, the induced electric fields will be relatively uniform over the extent of individual neurons. We therefore used the quasi-uniform assumption [45; 99], calculating the resulting extracellular potential along the z-axis, as $V_{EC} = E_{cn}z$. Here, E_{cn} is the electric field along the cortical normal direction (the z-axis of the local coordinate system), which we obtain directly from the lead field matrix of the New York head model [8; 52].

The New York head model takes into account the detailed geometry of the head, in addition to the very different electric conductivities of the white matter, gray matter, air cavities, cerebrospinal fluid, skull, and scalp, with conductivity values of 0.276 S/m for gray matter, 0.126 S/m for white matter, 1.65 S/m for CSF, 0.01 S/m for the skull, and 0.465 S/m for the scalp [52; 129]. This model is “pre-solved” through numerical finite element simulations (FEM), where the lead field matrix **M** has been calculated and made freely available (https://www.parralab.org/nyhead/; [52]). It was constructed through current input to each one of 231 electrodes, where the electric field was calculated and stored at about 75,000 locations in the brain. The original size of the lead field matrix is 231×75,000×3. It is often convenient to simplify this by assuming that dipoles are always oriented along the local cortex normal direction, reducing the size to 231×75,000. These matrix elements can thus be interpreted either as the electric field (unit V/m) along the cortical normal direction at any one of the 75,000 cortical locations in response to tES from any one of the EEG electrodes, or equivalently, as a map from a unit dipole at any one of the 75,000 locations (assumed to be oriented along the cortical normal direction) to the resulting EEG signal at any one of the 231 electrodes.”

4. Maybe I missed this in the manuscript, but since the RT is only applicable in the subthreshold regime, how far is it applicable in the context of an active neuron model with intrinsic spiking behavior? Here the intrinsic membrane potentials should frequently reach a non-linear regime.

This is a very interesting question that prompted us to go back to the drawing board, leading to a new result section (Sec 2.4 “Intrinsically active cells”) and a new figure (Fig 5).

As discussed under the “Limitations” section, the RT-based approach is not equally well-suited for all tasks, and indeed intrinsically active neurons might be an example of a scenario where the traditional approach is preferable.

However, there can be cases where the RT-based approach remains helpful for intrinsic spiking behavior. Consider neurons in the STN of the basal ganglia (Fig 5A), which are intrinsically active at about 30 Hz, even in the absence of input (Fig 5B). These neurons are an important target for deep brain stimulation, so understanding how they are affected by ES is clinically important. We simulated (through the traditional well-established approach) how the intrinsic spiking behaviour of a macaque STN neuron (from Miocinovic et al., 2006) would be affected by an ES (ES parameters were arbitrarily chosen to be 100 μA, 10 Hz, 500 μm away from the soma, Fig 5C). The spiking behaviour of the cell was substantially modulated by the ES (Fig 5D versus Fig 5B).

Since the behaviour of the membrane potential is clearly non-linear, the RT can not be used directly; however, the effect of an ES can still be estimated, by inserting the ES current (Fig 5C) into the soma of a passive version of the cell model, and calculating the resulting extracellular potential at the stimuli location (orange dot in panel A): Through the RT, this corresponds to the isolated ES-induced response of the somatic membrane potential ΔV_m of 2.1 mV (Fig 5E). We then introduce this effect into the active intrinsically spiking cell model as the somatic current input that in isolation would cause this ΔV_m. This gave rise to a very similar modulated activity as the traditional approach (Fig 5F versus Fig 5D).

The example above illustrates that the effect of the ES can—at least in this case—be isolated and then added to a non-linear model. This might for example be relevant for point neuron simulations of deep brain stimulation of the basal ganglia: Through the RT and single examples of biophysically detailed cell models, we can efficiently and easily get accurate estimations of how different cell-types at different locations are affected by an arbitrary ES. The effect at the network-level (with intrinsically spiking neurons) can be investigated by highly efficient point-neuron, or even mean-field network models.

We added this new text (section 2.4):

"2.4. *Intrinsically active cells*

We demonstrated that the RT-based approach works well in the approximately linear subthreshold regime (Fig 2), however, neurons are—due to their ability to fire action potentials—fundamentally non-linear entities. Some classes of neurons also exhibit intrinsic spiking activity, like in the subthalamic nucleus (STN) of the basal ganglia which is an important target for DBS therapy in patients with Parkinson's disease [75].

Note that even though the effect of ES on neural dynamics can be non-linear, the direct, isolated effect of ES on membrane potentials usually is linear: As in the traditional way of simulating, the ES enters as an additive term in the cable equation, $(1/r_i)\partial E_{||}(x, t)/\partial x$, which only depends on the electric field along the neurite $E_{||}$ and the cable properties, and not on the membrane potential. The ES-induced effect itself on the membrane currents is therefore linear and additive, even when the resulting membrane potential and network dynamics is not. The RT-based approach might therefore be able to isolate the linear effect of the ES itself on the membrane potential, which can then be added to a non-linear model.

To investigate this, we considered a macaque STN neuron (Fig 5A), which intrinsically spikes at around 30 Hz (Fig 5B). We introduced an ES with parameters arbitrarily chosen to have an amplitude of 100 μA at 10 Hz, located 500 μm away from the soma (Fig 5C). The spiking behavior of the cell was substantially modulated by the ES (Fig 5D versus

Fig 5B). Since the behaviour of the membrane potential is clearly non-linear, the RT can not be used directly; however, the effect of an ES can still be estimated, by inserting the ES current (Fig 5C) into the soma of a passive version of the cell model, and calculating the resulting V_e at the stimuli location: Through the RT, this corresponds to the isolated ES-induced response of the somatic membrane potential ΔV_m (Fig 5E). We then introduce this effect into the active intrinsically spiking cell model (instead of the ES) as the somatic current input that in isolation would cause this ΔV_m. This gave rise to a very similar ES-modulated activity as the traditional approach (Fig 5F versus Fig 5D).

The example above illustrates that the effect of the ES can——at least in this case——be isolated and then added to a non-linear model. This is relevant for point neuron simulations of deep brain stimulation of the basal ganglia: Through the RT and using biophysically detailed cell models, we can efficiently and easily estimate how different cell-types at different locations are affected by ES. The non-linear effect of the ES at the network-level (with intrinsically spiking neurons) can then be investigated by highly efficient point-neuron, or potentially even mean-field network models."

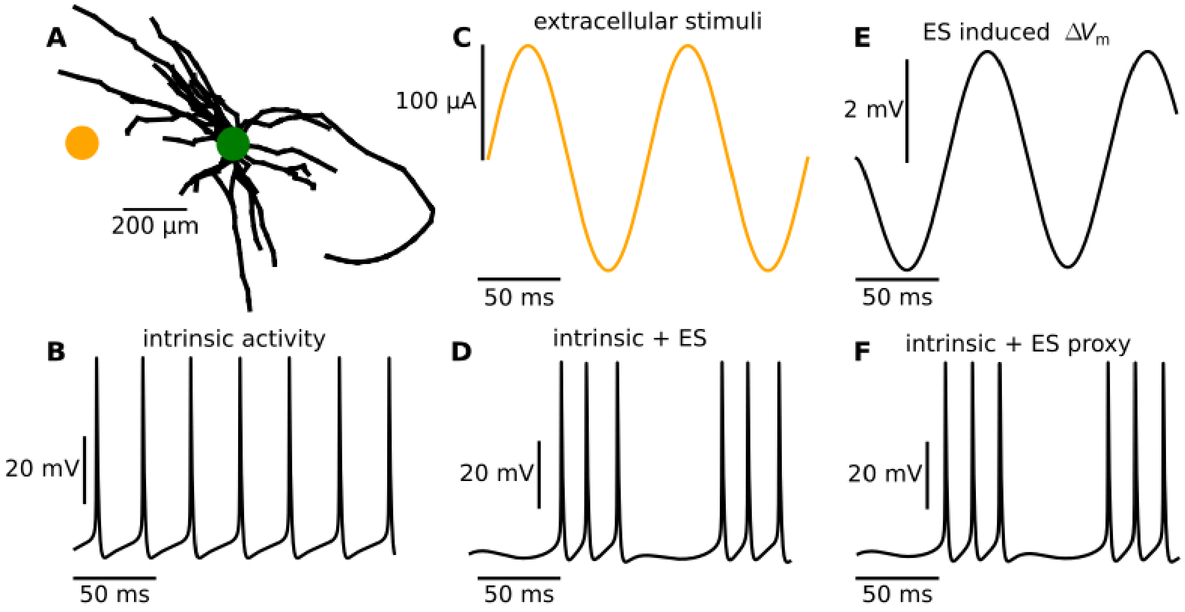

Fig 5: Intrinsically active cell model. A: Macaque STN neuron from Miocinovic et al. (2006) [43]. **B:** The intrinsic spiking activity of the model in the absence of input. **C:** An electric stimulation (location marked by orange dot in panel A) at 10 Hz with a current amplitude of 100 µA, 500 µm away from the soma of the cell model. **D:** The somatic membrane potential of the cell under the influence of the ES in panel C, simulated through the traditional approach. **E:** Through the RT, we can calculate the effect of the ES in isolation, by inserting the current in panel C into the soma of a passive version of the cell model, and calculating V_e at the ES location. The resulting amplitude of V_e was 2.1 mV. From the same simulation, we can calculate the input impedance of the model, and from this the equivalent somatic current amplitude of 67.6 pA needed to cause this 2.1 mV oscillation. **F:** The original active cell model is stimulated with an "ES proxy", that is, by injecting this current into the soma (amplitude 67.6 pA) designed to mimic the ΔV_m in panel E. This closely approximates the direct simulation (panel D).
