# Supplementary material for: Predicting neural responses to intra- and extra-cranial electric brain stimulation by means of the reciprocity theorem
Source: PLoS Comput Biol. 2025 Dec 15;21(12):e1013782. doi: 10.1371/journal.pcbi.1013782 (PMC12704905; doi:10.1371/journal.pcbi.1013782)
Supplement: Attachment — Submitted filename: ES_RT_Revision_2.pdf [file pcbi.1013782.s002.pdf]

Dear Editor;

We hereby resubmit our second revision of the manuscript “Predicting neural responses to intra- and extra-cranial electric brain stimulation by means of the reciprocity theorem” by Torbjørn V. Ness, Christof Koch, and Gaute T. Einevoll for publication in PLoS Computational Biology.

We would like to thank the editor and the three referees for their efforts in reviewing the manuscript. We’re happy that referees #1 and #3 are satisfied with our revisions and responded in detail (in blue) to the criticism of referee #2.

Changes in the manuscript from the first revision are marked as red.

Changes in the manuscript from this second revision are marked as orange.

With these changes we hope that the revised manuscript is found suitable for publication in PLoS Computational Biology.

Sincerely,

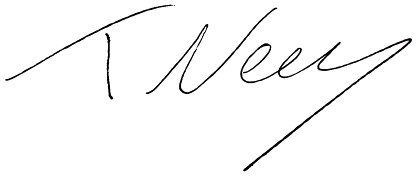

Torbjørn V. Ness

on behalf of the authors

Reviewer #1: The authors adequately addressed my points. I recommend this article for publication and am looking forward to more work from the authors demonstrating the practical use of RT in clinical neuroscience.

We are happy to hear this, and thank the reviewer for their effort.

Reviewer #2: Thank you for responding to my comments on the previous version. I do have a further few suggestions that I'd like the authors to consider.

1a) I still find the "novelty" aspect overstated and the new Figure 1 doesn't really help much, at least in my opinion. That said, I think there's a lot of value in the concept of reciprocity as a way to unite other findings in the literature. I would consider removing that Figure and slightly editing the introduction.

We are glad the reviewer sees the value in the suggested approach. The updated Figure 1 was added in response to Reviewer #1 (point 5); thus, we think it would be inappropriate to remove it. Furthermore, after discussing our work with other scientists during the last weeks, we found this figure very useful for conveying the main idea of the paper. We would therefore like to keep the figure.

We edited the introduction to focus less on the novelty and more on the usefulness of the reciprocity-based approach.
In line 10:
"This opens up ~~for entirely new applications of the RT, namely to~~ the possibility to directly study how the somatic membrane potential is affected by extracellular current stimulation in a straightforward manner."
In line 48:
"This is where the Reciprocity Theorem (RT) can ~~come to the rescue~~ be helpful."
In line 57:
"The RT is, however, also applicable if the current source is intracellular~~, with far-reaching consequences~~."
In Line 96:
"We demonstrate how this approach ~~leads to a better~~ aids in understanding of ES on different neural elements, …"

1b) I'd appreciate a more consolidated summary of the Lee et al. data: . i.e. something like "Lee et al. found X, which was unexpected b/c Y. Our model predicts X'..." I understood Figure 3 to be model vs. model predictions and the validation against empirical data sorta got lost in the shuffle.

This is a good point, and we agree that the validation against experimental data from Lee et al. (2024) was not well highlighted. We now moved this numerical prediction to a more natural location in the text, directly after the validation against the traditional modelling approach, line 126:
"We thus confirmed that the RT-based approach gives similar numerical predictions to the traditional approach in the approximately linear subthreshold regime. We can also compare these numerical predictions to measurements from the experimental literature: Lee et al. (2024) [28] recorded somatic V_m responses to ES of 100 nA, at a distance of 50 µm from the soma (in the frequency range 1–100 Hz) of about 0.3 mV. How does this compare to our results? At 10 Hz and a distance of 50 µm, a 1 µA current amplitude yields a V_m of about 3 mV (Fig 2B). Extrapolating linearly, the predicted V_m of an ES of 100 nA will be 0.3 mV, very similar to Lee et al. (2024) [28]. This serves as a qualitative check, confirming that our results are biologically plausible."

The results of Lee et al. (2024) were also used in a second context, where the focus was the reported surprising cell-type- and frequency-independent responses to ES. This is now better explained in the introduction, line 43:

"This difficulty is well illustrated by the recent results of Lee et al. (2024) [28], where even

single-cell subthreshold—and therefore presumably approximately linear—effects of ES in cortical slices are not easy to understand: They reported robust frequency-independent ES responses that were similar across cell-types, brain regions, and even species. This was surprising, because neurons are known to exhibit frequency-dependent behavior and have substantial cell-type specific variability in their morphological and electrophysiological properties. Neurons, therefore, have strong frequency- and cell-type dependent responses to intracellular current input."

3) I didn't mean to force the authors to totally disclaim applications to TI; I just thought it would be important to note that the mechanism may be different and thus over-estimate the effects.

Reducing the focus on temporal interference (TI) was previously suggested by Reviewer #3 (point 2). TI is, however, still mentioned at different locations in the manuscript.

4) The revised text helps a lot. Perhaps the root of my confusion was over what "sub-threshold" means. As I understand it, neurons embedded in an intact, awake brain generally sit within a few mV of their firing thresholds and so small changes in Vm can elicit "extra" spikes even if they aren't bringing a neuron all the way from it's equilibrium potential to firing. Would it be possible to include some rough numbers: "remains within the linear regime (~X mV from spike threshold)"?

The reviewer raises a valid point: it is, indeed, highly relevant to consider how accurate the RT-based approach is under physiologically more relevant, *in vivo*, conditions. This issue was already partially addressed in the first revision, where we demonstrated with the new Figure 5 ("Intrinsically active cell model") that the estimated linear isolated effect of ES accurately predicts the effect of ES on an intrinsically spiking neuron model. However, we agree that this could also be done more explicitly for other cell models. This is what we did in a new panel **F** in Figure 2:

Line 123:
"The relative error is 1.1%, 4.6%, and 12% for the pyramidal cell, the interneuron, and the axon terminal models, respectively, 1 mV below the spiking threshold, and reduces to 0.3%, 2.5%, and 4.0% at 5 mV below the spiking threshold (Fig 2F)."

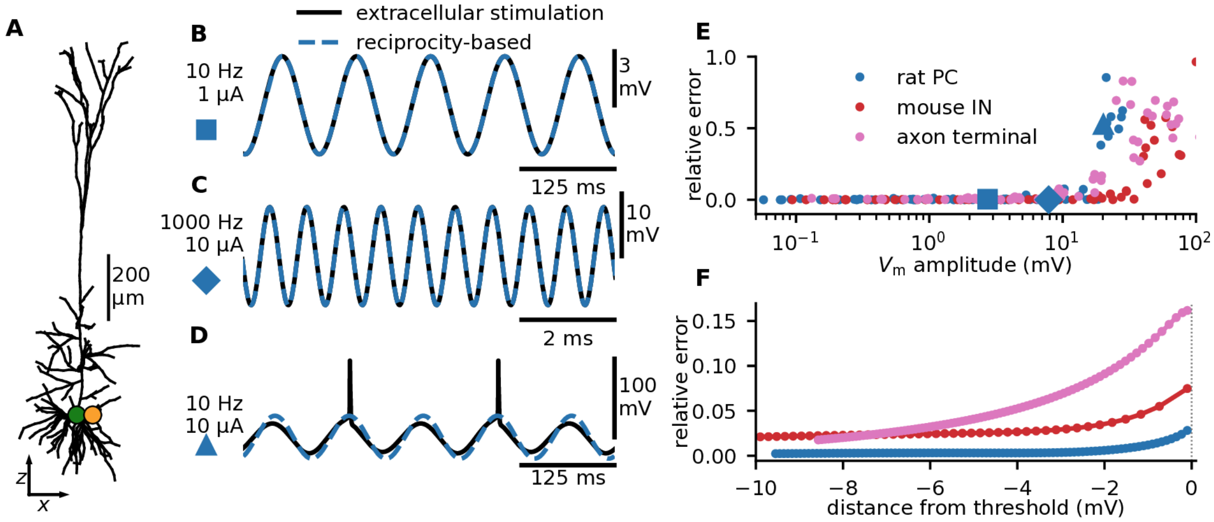

F: For each cell model 100 simulations were executed with small increments in the ES amplitude, where the range of amplitudes was chosen so that all models spiked for the highest amplitude. The highest subthreshold amplitude response in the target compartment was identified, and the spike threshold was defined as 0.1 mV above this value. The relative error of applying the RT-based approach is plotted versus the distance of the resulting membrane potential response from spiking threshold (only including subthreshold simulations). The ES was 50 µm outside the target compartment and had a frequency of 100 Hz.

6) Thanks. This is one of the examples that made the RT "click" for me.

Thanks, we agree that it is very appealing that one can use the RT to gain additional insights into ES from previous research exploring extracellular potentials.

I would add an additional caution here, since the authors mention predicting cell-type effects. While these are well-predicted by conventional models and in vitro experiments (e.g., Radman et al., 2009), I do not believe anyone has successfully identified them in vivo (Ozen et al., 2010; Krause et al., 2019, 2022; Johnson et al., 2020, etc). I don't think any of these are studies are necessarily wrong. Instead, network effects may swamp the effects of morphology: interneurons are less polarizable but more interconnected. Thus, the RT (or other isolated-neuron models) can tell you how stimulation directly affects a neuron but may not accurately describe the net effect on the brain. This point needs to be made very clearly!

We fully agree with the reviewer: To understand the ES, it is vital to study networks, as it is nontrivial to extrapolate from single-neuron to the whole system. However, the latter starts with the former. Understanding how single neurons respond to ES is, therefore, a necessary first step towards understanding network effects. Without an accurate

representation of cell-type specific effects of ES, network simulations are not built on solid ground. We reformulated the discussion to more clearly reflect this, line 566:

“Our study focuses on the single-cell level; however, to understand the effect of ES on the brain, it is vital to study network effects. In general, it is not feasible to predict these by extrapolating single-cell responses. However, accurate simulations of the network effects of ES must be built on an accurate representation of single cell effects.“

Reviewer #3: The authors addressed my comments very well. I support publication of the manuscript.

We are happy to hear this, and thank the reviewer for their effort.
